# Effects of Gamification on Performance and Subjective Listening Effort on a Spatial Release From Masking Task

**DOI:** 10.1044/2025_JSLHR-24-00794

**Published:** 2025-11-14

**Authors:** William J. Bologna, Katharine P. Buckheit, Katie Esser, E. Sebastian Lelo de Larrea-Mancera, G. Christopher Stecker, Frederick J. Gallun, Aaron R. Seitz

**Affiliations:** a Department of Speech-Language Pathology and Audiology, Towson University, MD; b Department of Psychology, Institute for Cognitive and Brain Health, Northeastern University, Boston, MA; c Center for Hearing Research, Boys Town National Research Hospital, NE; d Oregon Hearing Research Center, Oregon Health and Science University, Portland

## Abstract

**Purpose:**

Current theories of listening effort posit that motivation should influence a participant's level of effort and performance on a listening task. Gamification may be a means of manipulating motivation to test these hypotheses. This study compared a traditional and gamified version of a spatial release from masking test to determine if gamification yields better performance or subjective differences in listening effort compared to the traditional test.

**Method:**

Thirty-four adults (aged 20–47 years) with normal hearing completed a traditional and gamified version of a spatial release from masking test. The gamified test was themed as a spaceship race; correct responses made their ship go faster and revealed a pattern of game pieces that formed a path across the board. Both tests adapted the target-to-masker ratio based on performance to estimate threshold from a psychometric function. Subjective ratings of mental demand, fatigue, pace, enjoyment, frustration, and perceived success for each test were compared between two tests to evaluate perceived listening effort and characterize the participant experience.

**Results:**

Behavioral data indicated better thresholds in the gamified test than the traditional test. Participants reported more mental demand on the gamified test than the traditional test, but also more frustration and lower perceived success. The latter findings may have been driven by a propensity for participants to lose the race when the speech task was close to their performance threshold.

**Conclusions:**

Gamified testing is an effective way to improve performance and increase mental demand exerted by participants on speech-in-noise tests. Subjective ratings revealed that listeners' perception of success and frustration may have been biased by the outcome of the game, rather than the difficulty of the underlying speech task (identical for both tests). These results suggest that gamification may be a means of manipulating motivation to evaluate its effect on a speech-in-noise task.

**Supplemental Material:**

https://doi.org/10.23641/asha.30566462

Difficulty understanding speech in background noise is a common communication problem affecting virtually all hard of hearing individuals, as well as many individuals with normal hearing (Pang et al., 2019). Several clinical assessments of speech in noise are currently available, but widespread use of these tests is limited due to a variety of implementation barriers (Billings et al., 2023). In addition, the results of clinical tests of speech in noise are not always correlated with patient reports of communication difficulty in the real world (Souza et al., 2018). These limitations can lead audiologists to forego speech-in-noise testing altogether, which contributes to patient dissatisfaction with clinical care, particularly among patients with normal audiograms who report difficulty in noise (Mealings et al., 2020). As a result, there are significant unmet needs for assessment and rehabilitation of patients with speech-in-noise difficulty.

Individuals who report difficulty understanding speech in noise commonly describe the listening experience as effortful, tiring, or frustrating. In the interest of building the scientific knowledge around these dimensions of “listening effort,” Pichora-Fuller et al. (2016) proposed the Framework for Understanding Effortful Listening (FUEL). Briefly, FUEL describes effort as a capacity-limited system in which listeners spend mental resources to overcome the challenges of understanding speech in noise. The “cost” of spending these resources manifests as feelings of effortful listening, fatigue, and frustration. Intuitively, the demands of the listening task influence the effort experienced by the listener. For example, spectral degradation associated with noise-band vocoding has been shown to increase listening effort (Winn et al., 2015), whereas amplification has been shown to decrease listening effort for hard of hearing listeners (Hornsby, 2013). Another important dimension of FUEL is listener motivation. When a listener is highly motivated to understand speech, they are more willing to devote mental resources to overcome the challenges of background noise. This should, in theory, improve their speech understanding at the cost of increased listening effort. While the concepts of motivation, effort, and performance are intuitively intertwined, demonstrating these associations in experimental contexts has been identified as a priority area for future research (Pichora-Fuller et al., 2016).

FUEL extends the theory of motivational intensity (Brehm & Self, 1989) to listening effort. Motivation is a core component of a cost–benefit analysis that listeners may perform when allocating mental resources to a listening task (Eckert et al., 2016). If motivation is high, the expected value of successful communication outweighs the costs associated with effortful listening. If motivation is low, listeners may opt to conserve mental resources rather than engage in effortful listening. While these theories offer clear and testable hypotheses, experimental testing is complicated by the fact that motivation is hard to manipulate in a laboratory context. One approach has been to offer greater monetary rewards in certain experimental conditions to increase participant motivation to perform the task, but results from this approach are generally mixed. Increasing monetary rewards was shown to increase pupil dilation, a physiological manifestation of effort, but did not affect perceived effort or behavioral performance (Koelewijn et al., 2018, 2021). In other cases, greater monetary rewards increased perceived effort, but not behavioral performance or reaction times (Carolan et al., 2021). Increasing monetary rewards has also been shown to decrease fatigue in some studies (McGarrigle et al., 2024), but not in others (Gergelyfi et al., 2015). Overall, these results provide some support for notion that motivation can influence listening effort, but additional research with other methodologies would strengthen our understanding of these relationships.

Another approach to increase motivation on a listening task is through gamification. Gamification refers to the application of game design elements in contexts that are not traditionally a game (Deterding, 2012). In academic contexts, interest in gamification has increased over the past several years as a means of increasing student engagement in course content and motivation for assessments (Alomari et al., 2019; Domínguez et al., 2013; Huang et al., 2020; Koppitsch & Meyer, 2022; Taspinar et al., 2016). Similar applications of gamification have been reported in a variety of other contexts as well, including cognitive and auditory training (Green & Bavelier, 2015; Hamari et al., 2014; Kimball et al., 2013; Lelo de Larrea-Mancera et al., 2022; Whitton et al., 2014, 2017). The potential benefits of gamification are rooted in self-determination theory (Ryan & Deci, 2000), which posits that an individual's intrinsic motivation and behavior are mediated by their sense of competence, autonomy, and relatedness. Gamification is a means of addressing these psychological needs, thereby increasing the user's sense of motivation and performance on a given task (Gao, 2024). Despite these potential benefits, the application of gamification to speech-in-noise testing has been largely unexplored.

Viewed through the lens of self-determination theory, the potential to manipulate motivation on a speech-in-noise task through gamification becomes clear. Competence refers to an individual's sense of success or ability to perform the basic task set out for them. Understanding speech in noise is a universally challenging task, and frustration associated with speech-in-noise difficulty has been a longstanding dimension of hearing handicap (Ventry & Weinstein, 1982). Autonomy refers to an individual's level of control over their experience and the extent to which they are given meaningful choices to manipulate the environment. Traditional speech-in-noise testing involves a repetitive “call-and-response” format with highly controlled acoustic signals that offer minimal autonomy or control to the patient. These dimensions of self-determination theory can also be applied to listening effort and individuals with hearing loss; to the extent that hearing loss diminishes an individual's sense of competence and their autonomy to participate in a conversation, their motivation to engage may decrease, leading to social withdrawal (Matthen, 2016). The final dimension of self-determination theory is relatedness, or the extent to which an individual feels cared for and supported by others, and how much they can provide support to others through their autonomous choices. Perhaps, the greatest virtue of clinical speech-in-noise testing is its role in counseling and fostering a sense of relatedness between the patient and their audiologist (Billings et al., 2023). However, when clinical test results do not corroborate patient-reported difficulty, this sense of relatedness can be disrupted and further alienate the patient. Addressing these limitations of traditional testing is a primary goal of current efforts to produce gamified speech-in-noise tests. These tests have basic science applications for expanding our understanding of how motivation influences listening effort and speech understanding in noise, as well as potential for clinical translation if the benefits of gamification can be harnessed within a clinically appropriate test battery.

Previous work from our research group (Bologna et al., 2023) described a gamified version of the spatial release from masking (SRM) test (Gallun et al., 2013). The SRM test was selected for gamification because of its stable and consistent psychometric properties, simple application of binaural hearing cues to the listening task, and its sensitivity to effects of age and hearing loss (Gallun et al., 2013, 2015; Jakien et al., 2017; Marrone et al., 2008). In addition, the test uses stimuli from the Coordinate Response Measure Corpus (Bolia et al., 2000), which facilitates automated scoring necessary for independent gameplay. The gamified version of the SRM test included a visual puzzle component based on the computer game Pipe Mania (The Assembly Line, 1989). As participants responded to successive trials on the auditory task, the response buttons revealed straight and L-shaped pipe pieces that connected to form a path from the left side of the response grid to the right side. The intent of this initial investigation was to limit the scope of gamified elements and keep the task as close to the traditional SRM test as possible (as described by Gallun et al., 2013). This decision was motivated by the limited applications of gamification in audiology, and general caution on potential negative effects of gamification, which include distraction by gamified elements and misalignment of motivation to win the game rather than perform the task optimally (e.g., Katz et al., 2014). Results of this initial investigation were promising; no negative effects of gamification were observed over short testing durations, and potential benefits of gamification were observed over longer testing durations (Bologna et al., 2023).

Close inspection of the data from our initial study suggested potential learning effects over the course of the gamified runs, which lasted for 20–30 reversals of the adaptive track. The comparison data available in that study were from the traditional SRM test, which ended after the 10th reversal. Thus, there was not a clean comparison between the traditional test and the gamified test over long testing durations. The purpose of the current study was to evaluate effects of gamification by comparing a traditional and gamified test of SRM using long adaptive tracks, based on the hypothesis that longer durations of testing may be necessary to observe effects of gamification. This design also allowed us to evaluate the possibility that gamified testing accelerates task learning and improves internal consistency of responses within a test session. Speech-in-noise tests suffer from unwanted sources of variance within individuals, including learning effects and changes in motivation, effort, and fatigue throughout testing (Brungart et al., 2022). Gamification may be an effective way to minimize these unwanted sources of variance, as winning the game provides some intrinsic motivation to learn the rules and stay engaged in the task throughout testing. To evaluate these hypotheses, the current study tested participants for 30 reversals of the adaptive track in both the traditional and gamified tests. Trial-level data were fit to psychometric functions to test the predictions that performance thresholds would be better in the gamified test than the traditional test by the end of 30 reversals, and that the standard error of the fit of the psychometric function, which quantifies internal consistency of responses within a run, would be lower in the gamified test than in the traditional test.

In addition to changes in task performance associated with gamification, we were interested in subjective impressions of gamified testing among participants. Gamification was hypothesized to increase motivation, which should have predictable effects on self-reported dimensions of listening effort. To evaluate this hypothesis, participants provided ratings of both tests using a modified version of the NASA Task Load Index (Hart & Staveland, 1988), which has been used previously to evaluate subjective or self-reported listening effort (e.g., Bologna et al., 2013; Carolan et al., 2021; McGarrigle et al., 2024). The current application of this questionnaire included two questions related to listening effort (mental demand and fatigue), and several questions related to perceived usability of the game (pace of testing, perceived success, enjoyment, and frustration). The fatigue question replaced a question on physical demand from the original questionnaire to improve alignment with the dimension of listening effort. The enjoyment question was added to capture a new dimension of the testing experience that is not typically measured in other contexts. Differences in subjective ratings between tests were used to evaluate whether gamification influenced perceived listening effort, and to identify potential areas of improvement in future iterations of the game.

The current study aimed to answer the following research questions: (a) Does gamification yield better performance on an SRM task than traditional testing over a span of adaptive tracking lasting for 30 reversals? (b) How do subjective impressions of testing differ between traditional and gamified tests in dimensions of mental demand, fatigue, pace of testing, perceived success, enjoyment, and frustration? A new gamified test of SRM was designed with a more colorful story and visual effects, themed around a spaceship race. Performance and subjective ratings of the testing experience were compared between this gamified test (“Space Race”) and the traditional SRM task that the game was based on (SRM; Gallun et al., 2013). These results inform the continued iterative development of a gamified test of speech recognition in noise, with an ultimate goal of creating a gamified clinical assessment that captures a real-world listening experience in an engaging format and motivates patients to perform their best.

## Method

### Participants

The study sample included 34 adults, aged 20–47 years (*M* = 23.71, *SD* = 5.25) with normal hearing. Pure-tone air-conduction thresholds were measured using a GSI AudioStar Pro (Grason-Stadler, 2012) prior to the start of testing, and all participants had thresholds ≤ 20 dB HL at octave frequencies from 250 to 8000 Hz. All participants provided informed consent for the protocol and received course credit for their participation as approved by the institutional review board at Towson University (protocol #1494). The entire experimental protocol, including consent, audiometric testing, and completion of both the traditional and gamified tests, was completed in a single 1-hr session.

### Measures

**Figure 1.**
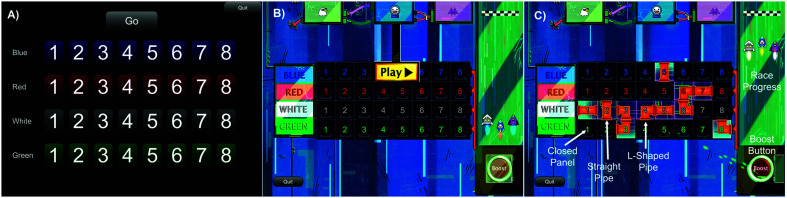
Response interface for the traditional and gamified tests. (A) The response screen for the traditional spatial release from masking test. (B) The Space Race response screen before the first trial has started. (C) The Space Race screen after several trials have been completed and the “boost” button is activated to prompt the participant to complete the circuit. Labels indicate a closed panel, straight pipe, and L-shaped pipe, along with the boost button and visual depiction of the race progress.

Both SRM tests (traditional and gamified) were implemented on a 10.2-in iPad (9th generation; Apple, 2021) running Portable Automated Rapid Testing (PART; Gallun et al., 2018) with Sennheiser HD 280 Pro headphones (Sennheiser, 2017). Target and masker sentences were from the Coordinate Response Measure Corpus (Bolia et al., 2000) and followed the structure “Ready [*call sign*] go to [*color*] [*number*] now.” Participants were instructed to listen for a sentence that started with the call sign “Charlie” and report the color–number combination spoken by that talker. Two to-be-ignored masker sentences were presented simultaneously using different call signs, colors, and numbers. The target and masker talkers were selected randomly from trial to trial from a set of three male talkers. The decision to randomize talkers was guided by the finding that speech recognition thresholds on this task are influenced by talker-specific variations in the stimuli (Brungart, 2001), and randomization controls for this unwanted variance while also ensuring that participants must use the call sign to identify the target, rather than any voice-specific cues. The response interface was a grid of four colors by eight numbers and was similar for the traditional and gamified test (see Figure 1), except that the gamified version had additional gamified visual elements (described below). Both SRM tests began with a single talker condition without maskers for practice and familiarization with the stimuli and testing procedure; these data were not analyzed. After the single talker condition, participants were tested in a co-located condition, where the target talker and two maskers were all perceptually located directly in front of the listener. Next, participants were tested in the separated condition, where the two maskers were convolved with head-impulse responses to simulate the perception of spatial locations at ±45°, as described in Gallun et al. (2013). Participants completed all conditions of the traditional or gamified test before completing the other test, and the order of the two tests (traditional and gamified) was counterbalanced across participants. In both tests, the co-located condition was tested before the separated condition.

Calibration was confirmed through PART with a second iPad running the NIOSH Sound Level Meter App and an iBoundary condenser microphone as described in Gallun et al. (2018). For both the traditional and gamified test, the target level was fixed at 65 dB SPL. Maskers started at 55 dB SPL (10-dB target-to-masker ratio [TMR]) in the traditional test, and at 57 dB SPL (8-dB TMR) in the gamified test. This difference in starting TMR between tests was unintentional, and the initial trial of the traditional test (with 10-dB TMR) was dropped from the analysis. In both traditional and gamified tests, masker level varied based on performance in 2-dB steps using a one-up/one-down adaptive staircase that ran for 30 reversals. Both tests also offered response feedback on each trial; feedback in the traditional test was a simple “correct” or “incorrect” displayed after each trial, and feedback on the gamified test was in the form of a special animation described below.

The gamified version of the SRM test, Space Race, used the same stimulus and response format as the traditional test with the added gamified context and visual elements of a spaceship race (see Figure 1B). In Space Race, the participant assumes the role of “Charlie” and is tasked with connecting a circuit inside the ship to make it go faster and win a race against other spaceships. The response grid used in the traditional SRM test was redesigned to look like a circuit with panels that open to reveal straight or L-shaped pipes for wiring. Target sentences from the Coordinate Response Measure corpus (e.g., “Ready Charlie, go to red six now”) were interpreted as instructions for which panels to open to complete a circuit from the left side of the screen to the right side. Response feedback was provided in the form of a short animation of green “energy” flowing through the panel after each correct response. Masker sentences were described as competing instructions being sent to the other ships, which are heard by Charlie due to a faulty communications system. In the separated condition, the participant was told that the crew chief has made an “adjustment” to the communication system so that the competing instructions sound like they are coming from the right and left. These instructions were designed to mirror the instructions provided in the traditional SRM test while keeping with the theme of the spaceship race.

After 12 trials, a message from the crew chief prompted the participant to press a “boost” button, which initiated a timed sequence without auditory stimuli where the participant had to click the remaining panels to complete the circuit from left to right (see Figure 1C). If the participant completed the circuit in time, such that the pipes formed a connected path from the left side of the response grid to the right side of the grid, they would win the race; if they did not complete the circuit in time, they lost the race. No data were collected during this timed “boost” phase. This aspect of the game was implemented to avoid potential visual cues that may emerge when most of the circuit puzzle was revealed, which may have allowed participants to use the visual pattern to identify the correct color–number response rather than relying on the auditory signals (see Bologna et al., 2023). After the race was over, a new circuit board was generated, and another race began with the adaptive track picking up where it left off at the conclusion of the previous race. Masker levels varied according to the same one-up/one-down adaptive staircase used in the traditional SRM test, and a single adaptive track was maintained from race to race until it reached 30 reversals. Participants completed 4–8 races per condition, depending on the number of trials needed to reach 30 reversals of the adaptive track. On average, the gamified test took 2–3 min longer to complete than the traditional test in both the co-located and separated conditions: traditional co-located = 3.46 min (*SD* = 0.66), gamified co-located = 6.06 min (*SD* = 0.98), traditional separated = 3.92 min (*SD* = 0.50), gamified separated = 6.84 min (*SD* = 1.19). The additional test time in the gamified test can be at least partially explained by the additional instructions in the game (messages from the crew chief explaining the race, faulty communication system, boost button), and the time required for the solve portion of each race (roughly 10–15 s per race).

After each SRM test (traditional and gamified), participants completed a short questionnaire, which prompted participants to rate their subjective experience with that task. Participants provided separate ratings on a 7-point scale for Mental Demand, Fatigue, Pace, Success, Enjoyment, and Frustration (see Table 1), with a rating of 1 indicating *not very*, 7 indicating *very*, and 2–6 representing five intermediate ratings in between these two descriptors. These subjective data provided insight into participants' perception of the two tests and revealed differences in motivation and user experience. Of note, a single questionnaire was completed after testing with both co-located and separated maskers. Thus, questionnaire data cannot be used to compare masker conditions, but only reflect differences between the gamified and traditional testing experiences.

**Table 1.** Subjective experience questionnaire domains and prompts.

Domain	Question prompt
Mental demand	How mentally demanding was the task?
Fatigue	How tired did the task make you feel?
Pace	How hurried or rushed was the pace of the task?
Success	How successful were you in accomplishing what you were asked to do?
Enjoyment	How enjoyable did you find this task?
Frustration	How frustrated, discouraged, irritated, stressed, and annoyed were you?

*Note.* Questionnaire on subjective experience. Each question was rated from 1 to 7, with 1 indicating *not very*, 7 indicating *very*, and 2–6 representing five intermediate ratings in between these two descriptors. Separate ratings were provided for the traditional and gamified test.

### Analyses

Previous work on a gamified test of SRM revealed changes in performance over the course of long testing durations, which complicates threshold estimation based on a specified set of reversals (Bologna et al., 2023). Considering the long testing durations employed here, efforts were made to maximize the use of the data and estimate a performance threshold holistically over the course of testing in each condition. On average, participants completed 50–60 trials in each condition at the point of the 30th reversal: traditional co-located = 51.8 trials (*SD* = 5.2), gamified co-located = 52.1 trials (*SD* = 5.11), traditional separated = 59.0 trials (*SD* = 6.44), gamified separated = 61.4 trials (*SD* = 8.76). The density of these data, which were centered around 50% threshold based on one-up/one-down tracks, facilitated estimation of the psychometric function to determine threshold. Whereas fewer data points were available at the tails of the psychometric function, all trials were included in the estimation to strengthen the regression analysis. Trial-level data were fit to a psychometric function using a generalized linear model implemented in R using the *glm* function (R Core Team, 2021; R package: *lme4*; Bates et al., 2015). The 50% correct point of the estimated function (TMR threshold) was extracted for each participant in the co-located and separated conditions for the traditional and gamified tests. This approach also allowed the standard error of the fit around the 50% correct point to be extracted to determine if gamification affected performance variability in the data. Supplemental Material S1 contains parallel analyses of thresholds estimated from the average of various subsets of reversals; results are nearly identical to those reported below.

TMR thresholds were analyzed with logistic regression using a linear mixed-effects model. The model specified TMR threshold as the dependent variable and estimated separate β coefficients for a number of independent variables and interactions with subject included as a random effect. The modeling process began with an omnibus model with all the following design-level factors and interactions included: Gamification (coded as 0 for traditional, 1 for gamified), Spatial Separation (coded as 0 for co-located, 1 for separated), and Run (coded as 0 for the first run, 1 for the second run). The order of traditional and gamified tests was counterbalanced across participants, but Run was included as a variable in the model to account for previously observed practice effects on the SRM task with spatially separated maskers (Bologna et al., 2023; Jakien et al., 2017). The omnibus model was evaluated using a stepwise process of factor elimination using model testing (Hofmann, 1997) to remove factors and interactions that did not significantly improve model fit. This resulted in a final model that best described the effects in the data with the fewest number of factors.

Subjective questionnaire data were analyzed separately for each of the six domains for differences between the traditional and gamified test. Bonferroni correction was applied to control familywise error rate across these six tests by setting the significance threshold to *p* < .0083. Ratings for each question were analyzed with separate two-way mixed effects analysis of variance (ANOVA) with gamification as a within-subject factor and test order as a between-subject factor. Test order (gamified first or traditional first) was included to account for potential context effects on the subjective ratings (Colle & Reid, 1998) but was nonsignificant in all cases.

## Results

**Figure 2.**
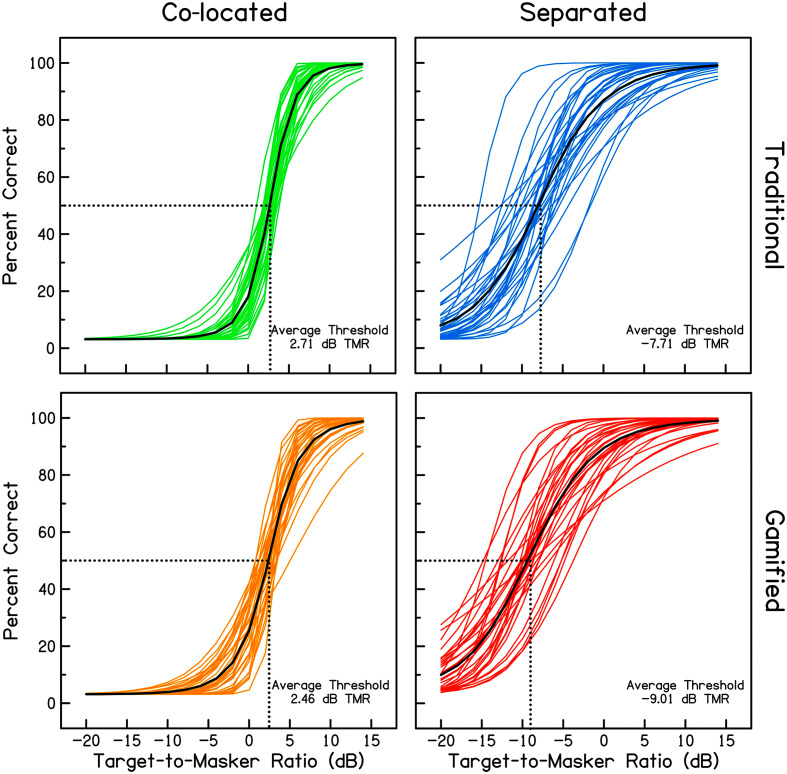
Individual psychometric functions in each condition are plotted as colored lines with the average psychometric function plotted in black. Co-located maskers are plotted in the left panels, and separated maskers are plotted in the right panels. The traditional test is plotted in the top panels and the gamified test is plotted in the bottom panels. In each panel, dotted lines indicate the average 50% threshold. TMR = target-to-masker ratio.

Estimated psychometric functions from each participant in each condition are depicted as colored lines in Figure 2, with the average function in black and dotted lines to illustrate the average TMR threshold. For both the traditional and gamified test, co-located maskers produced steeper and more consistent psychometric functions across participants as compared to separated maskers. The slope of each function was estimated over a 4-dB range around the 50% threshold and analyzed with a two-way repeated-measures ANOVA with factors of spatial separation and gamification. Results indicated a significant effect of spatial separation, *F*(1, 33) = 87.09, *p* < .001, such that slopes were steeper in the co-located condition than the separated condition. The effect of gamification also reached significance, *F*(1, 33) = 7.16, *p* < .05, indicating that the game produced slightly shallower slopes than the traditional test. The interaction between gamification and spatial separation was nonsignificant, *F*(1, 33) = 3.65, *p* = .065. The standard error of the fitted functions around the 50% correct point were analyzed with a similar two-way repeated-measures ANOVA. Results indicated a significant effect of spatial separation, *F*(1, 33) = 396.50, *p* < .001, such that the error of the fit for individual functions was greater in the separated condition than the co-located condition. No effect was observed for gamification, *F*(1, 33) = 1.16, *p* = .289, or interaction between gamification and spatial separation, *F*(1, 33) = 1.50, *p* = .230. These results suggest that gamification had little effect on the internal consistency of the data but that performance with separated maskers was more variable within a run than performance with co-located maskers.

**Figure 3.**
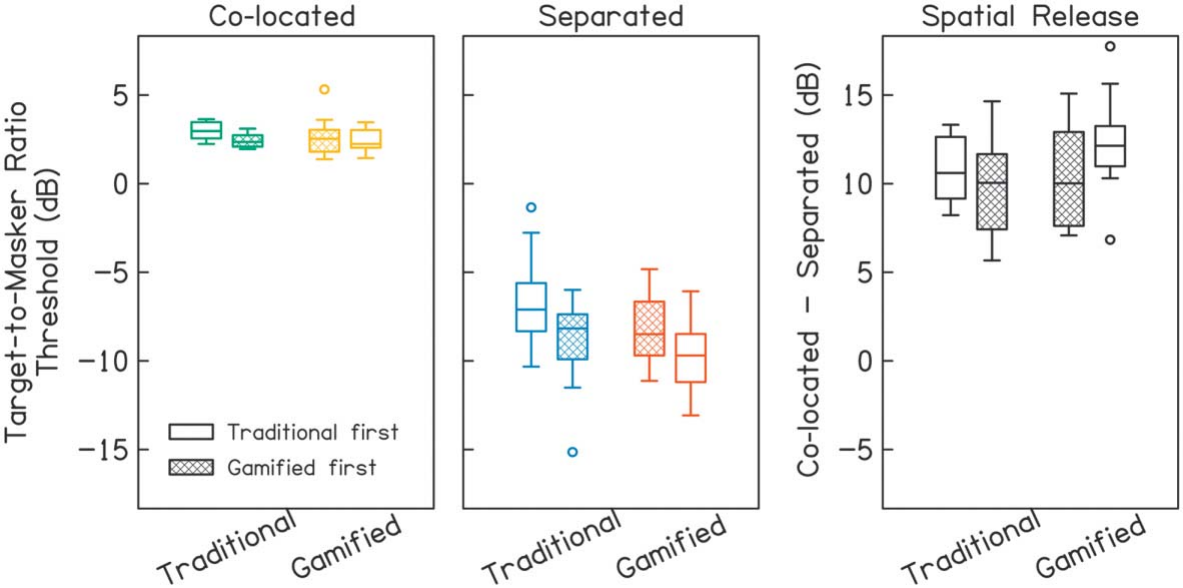
Target-to-masker ratio thresholds (dB) for the traditional and gamified tests with co-located maskers (left panel), spatially separated maskers (middle panel), and spatial release from masking (co-located – separated; right panel), with test order as a parameter (traditional first in unfilled boxes, gamified first in hatched boxes). For each distribution, the middle line indicates the median, the edges of the boxes indicate 1st and 3rd quartiles, whiskers indicate 10th and 90th percentiles, and the circles indicate outliers (more than 1.5 × interquartile range above or below quartiles).

The distributions of TMR thresholds in each condition are displayed in Figure 3 with test order as a between-subject parameter. Modeling results indicated significant main effects of Spatial Separation (χ^2^ = 235.23, *p* < .001) and Gamification (χ^2^ = 5.43, *p* = .020), as well as a significant interaction between Spatial Separation and Run (χ^2^ = 15.31, *p* < .001). All other effects and interactions did not significantly improve model fit (χ^2^ < 2.46, *p* > .117), in all cases, and were subsequently removed from the model. The final model, along with its factors and coding schemes, is summarized in Table 2 and can be expressed as follows:

$$\;\text{Threshold}\;\sim \;\text{Spatial Separation}+\text{Gamification}+\text{Spatial Separation}\times \mathrm{Run}+\left(\text{Subject}\right)$$ (1)

**Table 2.** Summary of linear mixed model predicting target-to-masker ratio (TMR) thresholds.

Factor	Coding scheme	Standard estimate (β)	*SE*	*t* value
(Intercept)	NA	2.94	0.30	9.74
Spatial Separation	0 = Co-located; 1 = Separated	−10.07	0.38	−26.55
Gamification	0 = Traditional; 1 = Gamified	−0.72	0.31	−2.33
Spatial Separation × Run	1 = Separated & Second Run	−1.75	0.44	−4.00

*Note.* Linear mixed model of TMR threshold with variable coding scheme, standard estimates, standard error, and *t* values for each significant effect and interaction term. NA = not applicable.

The effect of Spatial Separation on TMR threshold was robust (β *=* −10.07), indicating better TMR thresholds with spatially separated maskers. The effect of Spatial Separation also interacted with Run (β *=* −1.75), such that practice effects were more pronounced in the separated condition than the co-located condition; similar results were observed previously by Bologna et al. (2023). An effect of Gamification was also observed (β *=* −0.72), such that TMR thresholds were better in the gamified task than the traditional task; average thresholds differ by 1.3 dB with separated maskers and by 0.25 dB with co-located maskers. Of note, the effect of Gamification did not interact with Run, indicating that the effect of gamification was consistent regardless of test order.

**Figure 4.**
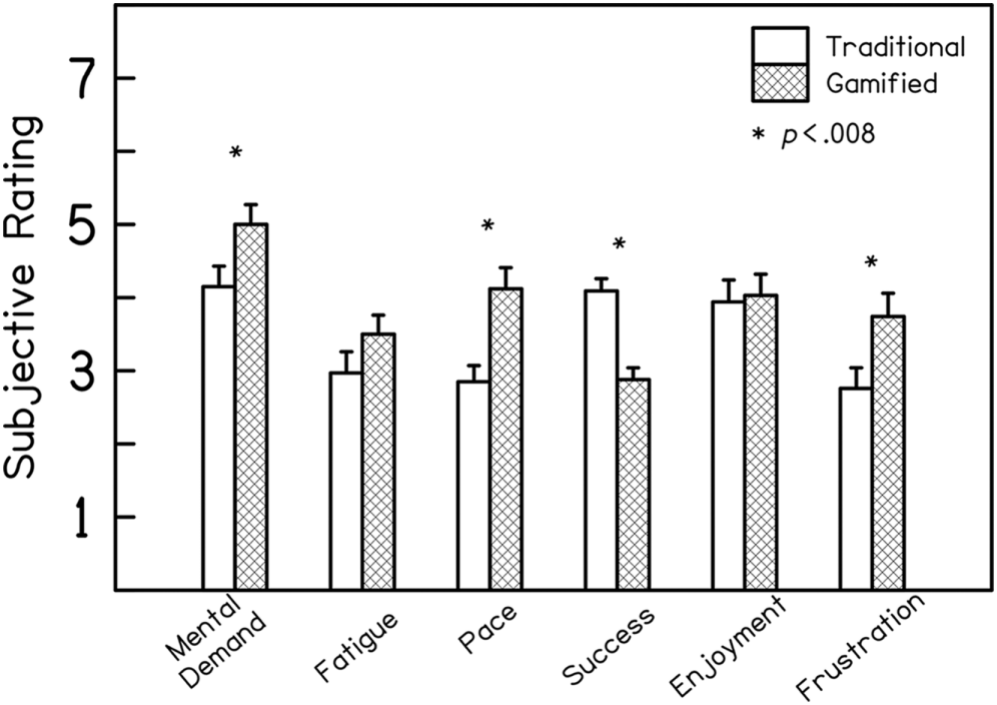
Subjective ratings of the traditional and gamified test (traditional in open bars, gamified in hatched bars). Higher ratings indicate greater mental demand, more fatigue, faster pace, greater success, greater enjoyment, and greater frustration. Asterisks indicate significant differences between the traditional and gamified tests; participants reported that the game required greater mental demand, had a faster pace, lower ratings of perceived success, and higher ratings of frustration.

Subjective ratings of mental demand, fatigue, pace, success, enjoyment, and frustration for the traditional and gamified tests are displayed in Figure 4. Statistical results indicated no effect of test order on any domain, and so data were collapsed across this variable in Figure 4 for clarity. In the domain of mental demand, participants reported significantly greater mental demand on the gamified test than the traditional test, *F*(1, 32) = 9.96, *p* = .003, η_p_^2^ = .069, with no effect of test order, *F*(1, 32) = 1.05, *p* = .314, or interaction with test order, *F*(1, 32) = 0.87, *p* = .357. No effects were observed in the analysis of fatigue ratings (gamification: *F*(1, 32) = 4.49, *p* = .042; test order: *F*(1, 32) = 0.03, *p* = .864; interaction: *F*(1, 32) = 1.80, *p* = .190). Participants reported a significantly faster pace of testing in the gamified test than the traditional test, *F*(1, 32) = 14.47, *p* = .001, η_p_^2^ = .167, with no effect of test order, *F*(1, 32) = 6.08, *p* = .190, or interaction, *F*(1, 32) = 0.24, *p* = .630. In terms of subjective success, participants reported significantly lower ratings of success on the gamified test than the traditional test, *F*(1, 32) = 32.89, *p* < .001, η_p_^2^ = .288, with no effect of test order, *F*(1, 32) = 1.04, *p* = .316, or interaction, *F*(1, 32) = 0.57, *p* = .457. No effects were observed in the analysis of enjoyment ratings (gamification: *F*(1, 32) = 0.18, *p* = .671; test order: *F*(1, 32) = 0.55, *p* = .466; interaction: *F*(1, 32) = 3.40, *p* = .075). Finally, subjective ratings of frustration were significantly greater for the gamified test than the traditional test, *F*(1, 32) = 7.93, *p* = .008, η_p_^2^ = .078, with no effect of test order, *F*(1, 32) = 2.99, *p* = .093, or interaction, *F*(1, 32) = 2.85, *p* = .101. Overall, these data indicate that participants reported greater mental demand, faster pace, lower perceived success, and greater frustration with the gamified test compared to the traditional test.

## Discussion

The current study is part of an ongoing line of research into the effects of gamification on speech-in-noise testing. The potential benefits of gamification include increased engagement in testing, higher motivation for performance, and a more positive overall impression of the testing experience. Effects of increased motivation on performance and listening effort have been theorized as part of FUEL, but additional empirical evidence is needed to further develop our understanding of these associations (Pichora-Fuller et al., 2016). This line of research on gamification contributes to this evidence base, while moving toward a long-term translational goal of creating a gamified clinic test that will capture the benefits of gamification, without compromising the clinical utility of the test results. In the current iteration of gamified testing, we observed positive effects of gamification on performance, and an associated increase in perceived mental demand, but an overall negative effect of gamification on the subjective experience and usability.

Contrary to our previous investigation of a gamified SRM test (Bologna et al., 2023), here, we observed a positive effect of gamification on performance. This difference in results is likely due to increased testing duration in the current study and an analysis approach that was better suited for the quantity of data that the increased testing duration provided. The most commonly reported benefits of gamification are in promoting prolonged engagement and motivation to perform well (Hamari et al., 2014; Huang et al., 2020), which require sufficient time on task to fully emerge. In Bologna et al. (2023), thresholds were estimated based on the average of the last six of 10 reversals in an adaptive track. This approach was adopted from the existing SRM task, based on Gallun et al. (2013), and reflects a typical design for a quick estimation of threshold in a psychoacoustic task. Here, thresholds were extracted from a full psychometric function estimated using a general linear model based on all trials in an adaptive track lasting for 30 reversals. Using these methods, a positive effect of gamification was observed, with lower thresholds in the gamified task than the traditional task with both co-located and separated maskers. As an additional step to evaluate our interpretation that longer testing duration affected the results, we estimated thresholds based on the average of reversals 4–10 in our long adaptive tracks using identical methods described in Bologna et al. (2023). An ANOVA of these threshold estimates did not reveal an effect of gamification, *F*(1, 33) = 3.55, *p* = .068, indicating that similar results would have been observed in these data using the same test length and threshold estimation approach used previously. This observation supports our interpretation that effects of gamification are more likely to emerge over longer testing durations.

Of note, the effect of gamification observed in this study did not interact with other variables in the experimental design or influence the fit of the psychometric function. The lack of an interaction with spatial separation indicates that the gamification effect was independent of masker type. This result is in line with the framework of gamification and self-determination theory (Gao, 2024), which propose that gamification effects should apply to any task, regardless of the underlying factors contributing to performance (like spatial separation of maskers). Additionally, there was no interaction between gamification and run, indicating that the gamification effect was distinct from a general practice effect observed here and in other studies using the SRM task (e.g., Jakien et al., 2017). Finally, gamification did not influence the internal consistency of participant responses, based on the standard error of the fitted psychometric functions. Results indicated greater variability in the fit of the functions for separated maskers than co-located maskers (expected finding based on greater variability in performance with spatial separation), but no effect of gamification on the fit of the function, indicating similar internal consistency of data for traditional and gamified tests. Gamification did have a small effect on the slope of the estimated functions, which were slightly shallower in the gamified test than the traditional test. Interpretation of this subtle effect is unclear at this time, and future research on gamified tests should evaluate slopes when possible to determine whether this effect is reproducible.

Responses on the subjective questionnaire corroborate the interpretation that improved performance associated with gamification reflects an increase in listening effort. Participants reported significantly greater mental demand on the gamified test compared to the traditional test, despite the fact the listening tasks were essentially identical. The inadvertent difference in starting TMR between the traditional and gamified tests was unlikely to affect these results, as the difference was very subtle and the additional data points were excluded from the analysis. FUEL posits that increased listening effort is often accompanied by increased listening fatigue (Pichora-Fuller et al., 2016), but this association was not observed in this study. While there was a trend toward greater subjective report of fatigue in the game than the traditional test, this comparison did not reach statistical significance. Of note, the duration of testing was typically less than an hour, and all the participants were younger adults. The association between listening effort and fatigue likely depends on the duration of effortful listening and, to some extent, on the age of the listener. Alternatively, it is also possible that gamified testing helps listeners stave off fatigue effects observed in repetitive laboratory listening tasks. Thus, the increased fatigue associated with effortful listening may be compensated, to some extent, by the increased engagement that participants experience when playing a game. Future research on gamified testing should explore these effects on fatigue using physiological methods that are better suited for quantifying fatigue than self-report (e.g., Gergelyfi et al., 2015). Other methods of evaluating listening effort in gamified tests are also warranted, as listening effort is an inherently multidimensional construct that is best described using a confluence of subjective, objective, and physiological measures (Pichora-Fuller et al., 2016).

The remaining items on the questionnaire allowed us to compare the participant experience with both tests and guide iterative improvements to the game's design. Participants reported poorer subjective perception of their own success and higher rates of frustration on the game compared to the traditional test. Remarkably, these negative perceptions of the game were not reflected in the self-reported level of enjoyment, which was nearly identical for the two tests. We attribute these findings to the fact that participants often “lost” the race in the gamified test. TMR adapted continually in a single one-up/one-down adaptive track for 30 reversals, and this tracking procedure converges quickly on the 50% correct point of the psychometric function (Levitt, 1971). To complete 30 reversals of the adaptive track, most participants had to complete 3–4 races, many of which were based on trials near their performance threshold. If the rate of correct trials is near 50%, then roughly half of the response tiles selected will not be part of the puzzle path, which makes solving the puzzle in the amount of time available very difficult. This may have also contributed to the subjective ratings of faster pace in the gamified test compared to the traditional test (which had no timed component). Ultimately, participants lost many of the races during testing. It is likely that they internalized these lost races as an indication of poor performance, leading to low ratings of perceived success and high ratings of frustration. Despite the negative experience, this finding has an important implication: participant self-perceived success seems to have been driven by the outcome of the race, rather than their actual performance on the listening task (recall that performance on the gamified test was better than the traditional test). Adjusting the difficulty of the race by slowing down the other ships at difficult TMRs would be a simple way to manipulate the race outcome without affecting the underlying listening task. Future gamified tests could leverage positive game outcomes (winning the race) to improve subjective impressions of success and potentially lower frustration, without affecting the difficulty of the listening task. Iterative improvements are a necessary component of game design, and current work is underway to address these issues as well as others.

### Future Directions

Future iterations of a gamified speech-in-noise test are already under development to address the issues with perceived success and frustration with the game outcome. Making the races easier to win can be achieved in a variety of ways, but the most straightforward is to include easier trials at the beginning of each race. The adaptive scan (Lelo de Larrea-Mancera et al., 2023) is an alternative approach to adaptive testing from the traditional up-down adaptive staircase that would fit this need. The adaptive scan method involves testing a series of tracks that progress in difficulty starting at a difficulty level above threshold and progressing to a predetermined stopping rule (e.g., 10 trials or three missed trials). After each track, the starting difficulty of the next scan is adjusted based on performance in the previous track to center the difficulty progression around the participant's threshold. This process continues for some predetermined number of scans, which are then used to calculate a threshold. The number of trials included in each scan could be set to match the number of trials in the race, such that each race begins a new scan, which starts at a difficulty level well above threshold. This would ensure that each race includes sufficient correct trials to allow participants to solve the puzzle without extreme difficulty. Testing with shorter scans rather than longer adaptive tracks also improves generalization of performance to other conditions, making the adaptive scan particularly well suited for gamified training and perceptual learning (Hochstein & Ahissar, 2002; Hung & Seitz, 2014).

Other game considerations are also being iteratively added to future work. Current games in development include more compelling storylines tied to more ecologically valid stimuli. While the Coordinate Response Measure corpus offers a closed set task that lends itself to a “game board” for the response interface, the structure and content of the sentences are not particularly engaging or meaningful. Current work in progress is using Bamford–Kowal–Bench sentences (Bench et al., 1979) with semantic and syntactic keyword foils as possible response options to retain the closed-set advantage of automatic scoring, while providing a broader and more meaningful semantic and syntactic structure to the stimuli and game themes. Different response paradigms are also being explored within the game context, shifting the behavioral assessment away from the “call-and-response” format and toward more ecologically valid speech response behaviors and measures of speech comprehension. These games also offer the participants more autonomy within the game context by giving them control over an avatar they use to navigate a space and “talk” to various nonplayer characters (NPC). Each NPC represents a target talker with masker configurations based on the position of maskers displayed visually in the game. Shifting control over the experience to the user will allow us to leverage additional benefits of gamification through the sense of autonomy, one of the pillars of self-determination theory (Ryan & Deci, 2000).

### Clinical Translation

In a clinical setting, where time is limited, speech-in-noise testing must be kept as brief as possible. Quick tests may not capture motivational and engagement aspects of communication that reflect real-world experiences, and our results suggest that additional trials may be necessary for these factors to emerge. The current iteration of Space Race took 2–3 min longer to complete per condition, compared to the traditional test. We attribute this difference to the additional thematic instructions that the game required, as well as time devoted to puzzle solving at the end of each race. While these features transformed the traditional test into a more engaging gamified experience, it came at the cost of additional test time. Thus, a balance must be struck between testing time and the sensitivity to motivation and other aspects of listening effort. One virtue of Space Race is that it is self-scoring and runs on an iPad; patients could play the game in the waiting room and provide high-quality data to the audiologist without any additional expenditure of clinical time.

As we move toward clinical translation of gamified speech-in-noise tests, additional research should focus on realistic patient populations of varying ages and degrees of hearing loss. Future work should determine the extent to which the increase in listening effort associated with gamification improves the correlation between test results and self-reported difficulty in real-world environments. Our current work on younger adults with normal hearing provides limited information on this hypothesis, as self-reported difficulty is presumably low among participants in our sample. Similarly, the frustration that participants reported in the game may, to some extent, reflect a meaningful dimension of real-world listening difficulties. The extent to which some degree of frustration is useful in a gamified context should be explored, as frustration in background noise is a commonly reported experience among hard of hearing listeners. Finally, gaming preferences may shift with age or generation (Wang, 2024), and additional usability work in older adults is necessary for clinical translation of these games to a typical audiological patient population. To the extent that gamification can capture motivational influences on speech recognition in noise, this approach may offer a new dimension to clinical testing.

## Conclusions

Gamified testing led to improved performance and increased mental demand on an SRM task, compared to the traditional laboratory test. These effects on performance and listening effort are consistent with an increase in participant motivation due to gamified features, suggesting that gamification may be a means of manipulating participant motivation on speech-in-noise tests. Participants self-reported lower levels of success and greater frustration in the game than the traditional test, and these perceptions may have been driven by the game outcomes, rather than their objective performance. Future work will address these limitations and continue exploring the effects of motivation on various aspects of listening effort and performance, while pursuing a parallel goal of developing of a clinically translatable speech-in-noise game.

## Data Availability Statement

Deidentified data are available from the corresponding author upon request. Traditional and gamified tests of spatial release from masking are also available for download upon request.

## Supplementary Material

10.1044/2025_JSLHR-24-00794SMS1 Supplemental Material S1 Statistical analysis of thresholds estimated by reversal averages.
