# Supplementary material for: Effects of Gamification on Performance and Subjective Listening Effort on a Spatial Release From Masking Task
Source: J Speech Lang Hear Res. 2025 Nov 14;68(12):6144–56. doi: 10.1044/2025_JSLHR-24-00794 (PMC12704924; doi:10.1044/2025_JSLHR-24-00794)
Supplement: Supplemental Material S1 [file JSLHR-68-6144-s001.pdf]

Supplemental Material S1. Statistical analysis of thresholds estimated by reversal averages.

In the main manuscript, thresholds were estimated based on the 50% correct point of psychometric functions, which were generated using generalized linear modeling. Another conventional approach to threshold estimation is based on the average of a subset of reversals at the end of the adaptive track. The current study used adaptive tracks lasting for 30 reversals, which complicates the decision of which reversals to use for threshold estimation. In order to fully explore the data, thresholds were estimated based on the average of all reversals, the average of the last 5 reversals (reversals 26-30), and the average of reversals 6-10 (similar to conventional short adaptive tracks). Average thresholds and standard deviations are summarized in the table below for each of these threshold estimations, with the psychometric function thresholds included for comparison. Average threshold differences across these estimates are generally less than 1 dB.

**All values = Mean (std. dev)**	Psy. Function	Average of all reversals	Average of reversals 26-30	Average of reversals 6-10
Traditional Colocated	2.71 (0.64)	2.74 (0.61)	2.74 (0.98)	2.62 (1.22)
Gamified Colocated	2.46 (0.91)	2.52 (0.89)	2.06 (1.37)	2.42 (1.19)
Traditional Separated	-7.71 (2.91)	-7.13 (3.03)	-8.01 (3.49)	-6.79 (3.83)
Gamified Separated	-9.01 (2.70)	-8.54 (2.77)	-9.60 (2.90)	-7.99 (3.97)

Three additional linear mixed effects models were generated from threshold estimates generated by averaging reversals following the same process described in the main text. The results are nearly identical to those reported in the manuscript for thresholds estimated from the psychometric functions. Full descriptions of these models with results tables are below.

Average of all reversals

In the model of thresholds estimated from all reversals, the effect of Spatial Separation on TMR threshold was robust, β = -9.48, indicating better TMR thresholds with spatially separated maskers. The effect of Spatial Separation also interacted with Run, β = -1.96, such that practice effects were more pronounced in the separated condition than the colocated condition; similar results were observed previously by Bologna et al. (2023). An effect of Gamification was also observed, β = -0.76, such that TMR thresholds were better in the gamified task than the traditional task; average thresholds differ by 1.41 dB with separated maskers and by 0.22 dB with colocated maskers. Of note, the effect of Gamification did not interact with Run, indicating that the effect of gamification was consistent regardless of test order.

**Factor**	**Coding Scheme**	**Standard Estimate (β)**	**Standard Error**	***t* Value**
(Intercept)	NA	3.01	0.31	9.72
Spatial Separation	0 = Colocated; 1 = Separated	-9.48	0.39	-24.47
Gamification	0 = Traditional; 1 = Gamified	-0.76	0.32	-2.39
Spatial Separation × Run	1 = Separated & Second Run	-1.96	0.45	-4.38

Average of reversals 26-30

In the model of thresholds estimated from reversals 26-30, the effect of Spatial Separation on TMR threshold was robust, β = -10.44, indicating better TMR thresholds with spatially separated maskers. The effect of Spatial Separation also interacted with Run, β = -1.53, such that practice effects were more pronounced in the separated condition than the colocated condition; similar results were observed previously by Bologna et al. (2023). An effect of Gamification was also observed, β = -0.98, such that TMR thresholds were better in the gamified task than the traditional task; average thresholds differ by 1.59 dB with separated maskers and by 0.68 dB with colocated maskers. Of note, the effect of Gamification did not interact with Run, indicating that the effect of gamification was consistent regardless of test order.

**Factor**	**Coding Scheme**	**Standard Estimate (β)**	**Standard Error**	***t* Value**
(Intercept)	NA	2.89	0.36	7.99
Spatial Separation	0 = Colocated; 1 = Separated	-10.44	0.46	-22.49
Gamification	0 = Traditional; 1 = Gamified	-0.98	0.38	-2.58
Spatial Separation × Run	1 = Separated & Second Run	-1.53	0.54	-2.86

Average of reversals 6-10

The model of thresholds estimated from reversals 6-10 differed somewhat from the other models reported here and in the main text. The effect of Spatial Separation on TMR threshold was similarly robust, $\beta = -10.44$, indicating better TMR thresholds with spatially separated maskers. However, the main effect of Gamification and the interaction between Spatial Separation and Run no longer significantly improved model fit (Gamification: $\chi^2 < .001$, Interaction: $\chi^2 = 3.02$, $p = .08$). Instead, the variance associated with Gamification was better explained by an interaction between Spatial Separation and Gamification, $\beta = -2.06$, indicating that the positive effect of gamification was only observed in with separated maskers. A three-way interaction between Spatial Separation, Gamification, and Run also significantly improved model fit, $\beta = -1.33$, indicating that the effect of gamification with separated maskers was larger for participants who completed traditional testing before playing the game. In general, the differences in these results from those reported in the main text are subtle and reflect reduced sensitivity to the effect of gamification at the point of the 10th reversal, as compared to other threshold estimation metrics.

**Factor**	**Coding Scheme**	**Standard Estimate (β)**	**Standard Error**	***t* Value**
(Intercept)	NA	2.52	0.35	7.15
Spatial Separation	0 = Colocated; 1 = Separated	-8.53	0.54	-15.94
Spatial Separation × Gamification	1 = Separated & Gamified	-2.06	0.79	-1.45
Spatial Separation × Gamification × Run	1 = Separated, Gamified, 2nd run	-1.33	0.92	-1.45
